# Mechanisms and Therapy for Cancer Metastasis to the Brain

**DOI:** 10.3389/fonc.2018.00161

**Published:** 2018-05-24

**Authors:** Federica Franchino, Roberta Rudà, Riccardo Soffietti

**Affiliations:** Department of Neuro-Oncology, University and City of Health and Science Hospital, Turin, Italy

**Keywords:** brain metastases, chemotherapy, neuroimaging, neuropathology, surgery, stereotactic radiosurgery, whole-brain radiotherapy, targeted therapy

## Abstract

Advances in chemotherapy and targeted therapies have improved survival in cancer patients with an increase of the incidence of newly diagnosed brain metastases (BMs). Intracranial metastases are symptomatic in 60–70% of patients. Magnetic resonance imaging (MRI) with gadolinium is more sensitive than computed tomography and advanced neuroimaging techniques have been increasingly used in the detection, treatment planning, and follow-up of BM. Apart from the morphological analysis, the most effective tool for characterizing BM is immunohistochemistry. Molecular alterations not always reflect those of the primary tumor. More sophisticated methods of tumor analysis detecting circulating biomarkers in fluids (liquid biopsy), including circulating DNA, circulating tumor cells, and extracellular vesicles, containing tumor DNA and macromolecules (microRNA), have shown promise regarding tumor treatment response and progression. The choice of therapeutic approaches is guided by prognostic scores (Recursive Partitioning Analysis and diagnostic-specific Graded Prognostic Assessment-DS-GPA). The survival benefit of surgical resection seems limited to the subgroup of patients with controlled systemic disease and good performance status. Leptomeningeal disease (LMD) can be a complication, especially in posterior fossa metastases undergoing a “piecemeal” resection. Radiosurgery of the resection cavity may offer comparable survival and local control as postoperative whole-brain radiotherapy (WBRT). WBRT alone is now the treatment of choice only for patients with single or multiple BMs not amenable to surgery or radiosurgery, or with poor prognostic factors. To reduce the neurocognitive sequelae of WBRT intensity modulated radiotherapy with hippocampal sparing, and pharmacological approaches (memantine and donepezil) have been investigated. In the last decade, a multitude of molecular abnormalities have been discovered. Approximately 33% of patients with non-small cell lung cancer (NSCLC) tumors and epidermal growth factor receptor mutations develop BMs, which are targetable with different generations of tyrosine kinase inhibitors (TKIs: gefitinib, erlotinib, afatinib, icotinib, and osimertinib). Other “druggable” alterations seen in up to 5% of NSCLC patients are the rearrangements of the “anaplastic lymphoma kinase” gene TKI (crizotinib, ceritinib, alectinib, brigatinib, and lorlatinib). In human epidermal growth factor receptor 2-positive, breast cancer targeted therapies have been widely used (trastuzumab, trastuzumab-emtansine, lapatinib-capecitabine, and neratinib). Novel targeted and immunotherapeutic agents have also revolutionized the systemic management of melanoma (ipilimumab, nivolumab, pembrolizumab, and BRAF inhibitors dabrafenib and vemurafenib).

## Introduction

Recent advances in chemotherapy and targeted therapies have improved survival in cancer patients. In this context of a better-controlled systemic disease, brain metastases (BMs) are emerging as a new challenge for the oncologist.

Brain metastases are the most frequent intracranial tumors: the incidence of newly diagnosed BMs is 3–10 times the incidence of newly diagnosed primary malignant brain tumors (1) and is still increasing. This trend could be explained by improvement in the quality of neuroimaging [magnetic resonance imaging (MRI)] and increased survival of patients with solid tumors (2).

In adults, lung (36–64%), breast (15–25%), and skin (melanoma) (5–20%) are the most frequent sources of BMs. Less frequent are cancers from colon-rectum, kidney, prostate, testis, ovary, sarcomas, and unknown tumors (3).

Brain metastases occur more frequently in patients with advanced disease. However, in some subgroups of patients, such as HER2+ breast cancer receiving trastuzumab, the brain represents now the first, often solitary, site of metastatic relapse (4).

The biology of BM remains poorly understood. Interactions between circulating tumor cells (CTCs) and blood–brain barrier (BBB) components are required. Some cytokines may act as CTC attractants and promote BM formation. BM development involves several steps (extravasation through non-fenestrated capillaries, local proliferation, and neoangiogenesis). Recently, clinical studies have detected specific genomic alterations in BMs but not in the primary tumor or extracranial metastases (5–7). How and when metastatic cells that spread to the brain evolve in a divergent way remains elusive: answering these questions could open the way for novel-specific therapies.

## Biology and Pathophysiology of BMs

Metastatization from systemic cancer requires several steps and complex genetic, epigenetic and biological changes in tumor cells, globally defined as “metastatic cascade,” beginning with detachment from the primary tumor and invasion of the surrounding tissue, intravasation into blood vessels and hematogenous dissemination, arrest in brain capillaries, and extravasation. Ultimately, neoplastic cells have to colonize surrounding tissue induce angiogenesis and proliferate in response to local growth factors (8). Those cells that survive find microenvironments that are conducive to their growth and development, also known as “niches”(9); they then form micrometastases that may, in turn, establish clinically significant lesions after a fairly variable period of dormancy, in which requirements for cell division are acquired (10).

Tumor cells have the capacity to evade growth suppressors and inhibitors of cell proliferation *via* mechanisms that include the resistance of apoptosis by overexpression of Bcl-2, Bcl-xL and downregulation of pro-apoptotic Bax and Bim. Through a phenomenon called epithelial–mesenchymal transition, activated by intrinsic (gene mutations) or extrinsic factors (growth factor signaling), epithelial tumor cells can de-differentiate, migrate to a distant focus, survive to apoptosis, disseminate, and then re-differentiate to the original cell (11, 12). Activation of cells in the adjacent stroma (endothelial cells, cancer-associated fibroblasts, pericytes, and leukocytes) *via* paracrine signaling with pro-tumorigenic factors (transforming growth factor beta, hepatocyte growth factor, epidermal growth factor, fibroblast growth factor, and IL-6) sustain tumor growth, enhancing genomic instability and epigenetic dysregulation (8, 13, 14).

Invading tumor cells show a downregulation of proteins preserving structural tissue integrity, such as E-cadherins, integrins, and catenins, lose cell–cell adhesion, secrete proteolytic enzymes that degrade the epithelial basement membrane, penetrate the endothelial basement membrane of vessels, and enter the circulation. Tumor cells, which arrest in capillary beds adhering to the endothelium of target tissue, behave like macrophages, creating pseudopodia and penetrating the cell–cell junctions, and then gain access to the tissue parenchyma by activating angiogenic programs to develop a new vascular supply. Circulating cancer cells attract platelets because of their expressed surface tissue proteins, which protect them from the immune system (15, 16).

The BBB is a functional and anatomic barrier, which plays a central role in interacting with brain microenvironment and influencing metastatic colonization. Several components can be subjected to adaptions by metastatic tumors to breach this barrier. Studies have found a role of cell–cell adhesion factors, including cyclo-oxygenase 2, heparan-binding epidermal growth factor, and alpha-2,6 sialyltransferase (ST6GALNAC5). As the tumor cells adhere to the BBB, infiltrative and transmigratory processes allow the tumor cells to breach the BBB. Later, the tumor cells use the inflamed brain microenvironment as a *niche*. Tumor cells interact with activated microglia and astrocytes, which provide support for neuronal function (17). Studies have found that metastatic tumor cells secrete cytokines involved in the MAP kinase pathway: the hyperactivation of this pathway permits breast cancer cells to overexpress MMP2, a proinflammatory enzyme that helps in tissue remodeling and angiogenesis. Metastatic lung cancer cells have been found to produce IL-8, macrophage inhibitory factor, and plasminogen activator inhibitor 1. These factors activate astrocytes to produce growth factors (IL-6, IL-1B, and tumor necrosis factor), resulting in a perpetuation of cancer cell growth in the neural niche (18). Activation of VEGF, Notch pathways and secretion of BMP-2 (a growth factor that differentiates neuronal stem cells into astrocytes) sustain neoangiogenesis in BMs and tumor–astrocyte interactions (19).

Tumor-driven activation of the mitogen-activated protein kinase (MAPK) and AKT pathways promotes endothelin-driven astrocytic survival and upregulate the antiapoptotic genes BCL2L1, GSTA5, and TWIST1 (20). Tumor cells have also been observed to possess double-stranded DNA repair mechanisms, which protect from reactive oxidative species that are the primary mechanisms of microglial killing of tumor cells. Tumor metastatic cells activate mechanisms in the brain to escape immuno-surveillance [recruitment of myeloid-derived suppressor cells, reduced expression of transporter associated with antigen processing 1 (TAP1), thereby reducing the effect of T-cell-mediated cell death] (21).

Metastatic cells can also take advantage of other mechanisms in the neural niche, such as nerve growth factor–tropomyosin receptor kinase B interaction on breast brain metastatic cells amplifying oncogenic signaling (22). A subset of metastatic breast cancer tissue has been observed to possess an upregulation of GABA receptors and transporters compared with their primary tumor tissue counterparts: after transporting synaptic GABA into their own cytosol, it is converted to NADH in the cellular mitochondria enhancing energy production, cellular respiration, and finally metastatic cell function and survival (23).

Understanding the mechanisms of metastatic cell invasion and survival within the neural niche could elucidate solutions for metastatic cancer prevention and cure.

## Diagnosis: Clinical and Neuroimaging Features

Intracranial metastases are most frequently diagnosed in patients with an established primary site of malignancy (metachronous presentation). To a lesser extent, BMs are discovered at the same time of primary tumor (synchronous presentation in up to 30%) or may be the initial presentation as an occult malignancy (precocious presentation) in up to 10% of patients (24).

Intracranial metastases are symptomatic in 60–70% of patients with neurologic symptoms including headache (40–50%), focal neurological deficits (40%), and seizures (15–20%). Impaired cognition and altered mental status are frequent in patients with multiple metastases and/or increased intracranial pressure. Another 5–10% of patients present with acute “stroke like” symptoms due to an intratumoral hemorrhage (especially in melanoma, kidney cancer, and choriocarcinoma). However, symptoms and signs at presentation can be subtle. As a general rule, BMs should be suspected in any patient with known systemic cancer in whom new neurologic findings develop (25).

In both asymptomatic and symptomatic patients, imaging of the brain has a primary role in the diagnosis and is important for subsequent patient management (Table 1).

**Table 1:** Comparison of neuroimaging techniques in diagnosis of BMs.

Technique		Advantages	Disadvantages
CT		– First-line approach – Fast information on intracranial hemorrhage, herniation, mass effect, and hydrocephalus – Useful in non compliant patients	– Low resolution and low sensibility for differential diagnosis
MRI (T1/T2/FLAIR sequences)		– Major resolution – Better lesion characterization and localization (cortical/subcortical)	– Difficult resolution of calcifications – Longer time for image acquisition and processing
MRI with Gad		Better characterization	– Longer time – Allergic reactions to Gad
Advanced MRI	Spectroscopy	Useful in DD glioma vs BM	– Longer time
	Perfusion	Useful in DD HGG vs BM (rCBV value in peritumoral FLAIR hyperintense region)	– Longer time – Variable target definition for measurement – Unclear rCBV cutoff values in DD HGG vs BM
	DTI images	– Evaluate the structural organization of white matter tracts and spatial relationships with brain lesions – Useful in surgical planning	– Low specificity
	DWI and ADC images	– DD HGG/BM and abscesses (restricted diffusion with DWI+ is more typical in abscesses) – DD HGG/BM edema in peritumoral region (higher ADC signal in BM) – DD PCNSL/HGG and BM (DWI + ADC maps)	– Longer time
PET-CT	18F-FDG PET	– Metabolic and functional information	– Low specificity in DD with others brain lesions (inflammatory disease, abscesses, and granulomatous lesions) and other tumors (HGG)
	Amino acids PET	– More useful for the differentiation of tumor and non-tumoral processes, as tumors have significantly higher uptake	– Increased uptake in acute inflammatory lesions – Not sufficient discrimination from HGG and some non-neoplastic lesions

*CT, computed tomography; MRI, magnetic resonance imaging; Gad, gadolinium; DTI, diffusion tensor imaging; DWI diffusion-weighted imaging; DD, differential diagnosis; BMs, brain metastases; HGG, high-grade glioma; rCBV, relative cerebral blood volume; ADC, apparent diffusion coefficient; PET, positron emission tomography; 18F FDG, 18F-fluorodeoxyglucose; FLAIR, fluid attenuated inversion recovery*.

Computed tomography (CT) can provide information on intracranial hemorrhage, herniation, mass effect, and hydrocephalus. MRI with intravenous contrast is preferable for the greater sensitivity than CT, particularly for lesions in the posterior fossa or multiple punctate metastases (26). There are no specific features on MRI that characterize BMs; however, a peripheral location, spherical shape, ring enhancement with extensive peritumoral edema, and multiple lesions suggest a metastatic disease (27).

Differential diagnosis (DD) includes primary brain tumors [high-grade gliomas (HGGs), primary CNS lymphomas] and non-neoplastic conditions (abscesses, infections, hemorrhages, subacute infarcts, demyelinating diseases, granulomatous diseases, and radiation necrosis).

Advanced neuroimaging techniques have been increasingly used in the detection, treatment planning, and follow-up of BMs. The most frequently used techniques include MR proton spectroscopy (MRS), MR perfusion, diffusion-weighted imaging (DWI), and diffusion tensor imaging (DTI). MRS more often shows a lower choline to creatinine ratio in BMs than in HGGs (28). When employing MR perfusion (dynamic contrast-enhanced), although the relative cerebral blood volume (rCBV) ratio may not be reliable for a differentiation of the enhancing portion of HGGs from metastases, the evaluation of the peritumoral fluid attenuated inversion recovery (FLAIR) hyperintensity has shown lower rCBV in case of metastases compared with HGG: this is likely due to the presence of infiltrative cells and neoangiogenesis in HGGs, while metastatic lesions are surrounded by pure vasogenic edema. Peritumoural perfusion-weighted imaging can assist in preoperative differentiation between a glioma and a solitary metastasis, but to date there is no a definite cutoff value useful for radiological DD (29). In addition, evaluation of the DSC (dynamic susceptibility contrast MRI) perfusion signal changes over time in the contrast-enhancing mass has shown that metastasis has a vascular permeability or “leakiness” of contrast which is higher compared with an HGG but lower compared with PCNSL. DWI evaluates the mean diffusivity of water molecules in a given region of brain parenchyma: highly cellular tumor results in a “restricted diffusion” pattern, with hyperintensity on highly diffusion-weighted DWI sequences and lower signal intensity on apparent diffusion coefficient (ADC) images. Because of the various degrees of cellularity in different metastatic tumor types, DWI is highly variable (30). ADC can be helpful in distinguishing the peritumoral edema surrounding metastases, which usually demonstrates higher ADC signal, from the non-enhancing peritumoral area of HGGs, which demonstrates relatively lower ADC values (31). Diffusion-weighted MR imaging may be useful in the diagnosis of ring-enhancing lesions: restricted diffusion is more typical in abscesses compared with unrestricted diffusion in necrotic metastases or glioblastomas, but the findings are not specific (32). DTI is an MRI technique, which is able to evaluate the structural organization of the brain, particularly white matter tracts. It is useful in surgical planning to localize the relationships of a metastasis to the major white matter tracts. DTI has been also employed to differentiate metastases from HGGs (33, 34).

The capability of 18F-fluorodeoxyglucose (FDG) positron emission tomography (PET) in differentiating BMs from HGGs is limited, since a considerable overlap of standardized uptake values (SUV_max_) exists. 18F-FDG PET also has limited specificity for distinguishing metastases from non-neoplastic lesions, such as brain abscesses, demyelinating tumefactive (“tumor-like”) lesions, fungal infections, and neurosarcoidosis due to the increased glucose metabolism in inflammatory tissues (35).

Amino acid PET seems more useful for the differentiation of tumor and non-tumoral processes, as tumors have significantly higher uptake than non-neoplastic tissues. However, a moderate increase of uptake can also be seen in acute inflammatory lesions. In conclusion, in BMs, both 18F-FDG and amino acid PET do not provide sufficient discrimination from high-grade glial tumors and some non-neoplastic lesions (36, 37). Overall, no advanced imaging techniques can reliably identify the nature of an enhancing brain lesion, and hence histopathological analysis remains the gold standard.

A tissue diagnosis by biopsy should then be considered in patients with either unknown primary tumor or absent well-controlled systemic cancer, especially if a long interval has elapsed since the initial cancer diagnosis, or, seldom, in patients with active systemic cancer when the radiographic appearance is highly atypical and life expectancy is not too short.

When a brain mass is discovered on MRI and there is no prior history of cancer, in most cases the primary tumor is located in the lung (38, 39): thus, chest CT is recommended, while CT of the abdomen only occasionally shows an unsuspected cancer. Further search for a primary tumor is almost never fruitful in asymptomatic patients (40). Whole-body FDG PET is a sensitive tool for detecting a “probable” primary tumor by visualizing foci of abnormal uptake, more often in the lung (41, 42), but the specificity in differentiating malignant tumors from benign or inflammatory lesions is relatively low.

Regarding BMs from an undetected primary site after the first investigations, serial CT scans of the thorax during follow-up in asymptomatic patients can discover the primary tumor (a non-small cell lung carcinoma in the majority), but few patients only benefit in terms of survival from early detection and treatment (43). However, there are no data dealing with this issue in molecular subgroups with available targeted therapies [i.e., EGFR mutated or anaplastic lymphoma kinase (ALK)-rearranged tumors].

In patients with BMs, a CSF examination is not indicated, unless there are coexistent symptoms or signs or neuroimaging findings that suggest an associated leptomeningeal carcinomatosis.

## Diagnosis: Neuropathology

Routine hematoxylin–eosin stain of biopsy specimens usually allows the distinction of metastases from malignant gliomas, meningiomas, lymphomas, and more rare entities. Immunohistochemical markers may be useful for a further characterization of the tumor type.

The immunohistochemical expression of glial cell markers, such as glial fibrillary acidic protein or oligodendrocyte transcription factor (OLIG2), suggests a glial tumor.

Usually, BMs mimic the histological appearance of the primary tumor, but sometimes with a lesser degree of differentiation. Overall, the vast majority of BMs are adenocarcinomas. The presence of glandular architecture and mucous allows the distinction of an adenocarcinoma from a small or non-small cell carcinoma with neuroendocrine differentiation, squamous carcinoma, or unspecified non-small cell carcinoma (44). Apart from the morphological analysis, the most effective tool for characterizing a BM is immunohistochemistry (45).

Lung cancers are the most common cause of BM. The brain tropism of non-small cell lung cancer (NSCLC) is higher for adenocarcinoma (54.8%) and poorly differentiated carcinoma (31.7%) than for squamous cell carcinoma (46). Pulmonary adenocarcinomas express cytokeratin 7 in the absence of expression of cytokeratin 20 (CK7+/CK20− phenotype). However, most adenocarcinomas from breast or other origins behave similarly, thus other markers are needed (47). Thyroid transcription factor (TTF1) is a marker of lung and thyroid origin, and is expressed in 80% of primary non-mucinous lung adenocarcinomas. TTF1 is also expressed in 90% of small cell and 50% of non-small cell neuroendocrine pulmonary carcinomas, thus the pathologist should search for the expression of neuroendocrine markers (CD56, chromogranin A, and synaptophysin) and pancytokeratin AE1/AE3. Focal expression of TTF1 has been described in breast carcinoma, while 20% of primary pulmonary adenocarcinomas do not express TTF1. Anti-Napsin A expression may help to identify a small fraction of these TTF1-negative adenocarcinomas (48). A molecular analysis searching for molecular abnormalities should include mutations of epidermal growth factor receptor (EGFR), Kirsten rat sarcoma viral oncogene homolog (KRAS), BRAF, human epidermal growth factor receptor 2 (HER2), c-MET abnormalities, translocations of ALK, rearranged during transfection (RET), or repressor of silencing 1 (ROS1).

When a breast cancer metastasis is suspected, the expression of hormonal receptors [estrogen receptors (ERs) and progesterone receptors (PgR)] must be investigated, although their expression is not specific as observed in ovarian, endometrial adenocarcinomas, and bronchopulmonary adenocarcinomas (47, 49). Furthermore, breast cancers frequently express mammaglobin, gross cystic disease fluid protein-15 (GCDFP15), and trans-acting T-cell-specific transcription factor (GATA3) (50). An immunohistochemical overexpression of HER2 has prognostic and therapeutic significance (51). “Triple negative” breast cancers (absence of expression of HER2, ER, and PR) have the highest risk of BM and a poor prognosis.

The diagnosis of BM from colorectal (CRC) adenocarcinoma is often done based on the morphological appearance and is supported by the immunohistochemical profile (CK20+/CK7−). The expression of CDX2, a transcription factor expressed in the nuclei of intestinal epithelial cells, also favors the diagnosis, such as the finding of an activating mutation of the RAS genes. In CRC adenocarcinoma KRAS mutations are found in approximately 40% of patients, resulting in the activation of the RAS/RAF/ERK pathway, rendering EGFR inhibitors ineffective (52–54). KRAS and neuroblastoma RAS viral oncogene homolog (NRAS) are closely related, and mutations tend to be mutually exclusive. Few data are available on the molecular subsets of BM from CRC adenocarcinoma. In addition to RAS, activating mutations in the BRAF gene have been found in 8–10% of CRC cancers, and they are almost always mutually exclusive with KRAS mutations (55–57). They are typically associated with right-sided tumors, high-grade mucinous histology, high frequency of lymph node and peritoneal metastasis, and microsatellite instability (58, 59).

The diagnosis of squamous cell carcinoma is based on conventional histology (presence of intercellular bridges and keratinization). The immunohistochemical expression of cytokeratins 5/6 or p40 (more specific than p63 which is expressed in 26–65% of adenocarcinomas) can confirm the epidermoid differentiation (50, 60): however, no marker can specifically determine the primary site.

Other markers of squamous differentiation are CK34bE12 and desmocollin-3, while TTF1 is not expressed. The coexistence of morphological and immunohistochemical features of carcinoma and adenocarcinoma suggests the possibility of an adenosquamous carcinoma of pulmonary or gynecological origin.

Most of the patients with BM from an unknown primary site can be diagnosed by searching for mucins and TTF1 and p40. When a primary breast cancer is suspected, TTF1 and GATA3 are the most relevant markers (50). CK7 and CK20 are less helpful, since many cancers with brain tropism, such as NSCLC or breast cancer, have a CK7+/CK20− phenotype.

## Molecular Biology

In BM, molecular alterations do not always reflect those of the primary tumor (Table 2). In BM of NSCLC, fibroblast growth factor receptor 1 (FGFR1) amplifications are found in 19% of squamous cell carcinomas, and in 15% of adenocarcinomas. A positive correlation of ALK and FGFR1 gene amplification status exists in BMs (61). Discoidin domain-containing receptor 2 (DDR2) mutation was reported in 1–4% of Sq-NSCLC, and its sensitivity to dasatinib inhibition was demonstrated both *in vitro* and *in vivo* (62).

**Table 2:** Neuropathological and molecular markers of brain metastases.

Morphology	Histochemistry and IHC		Molecular biology	Standard detection method	Targeted therapy
Undifferentiated tumor	Desmin	Sarcoma			
	HMB45 MelanA	Melanoma	BRAF/NRAS mutations	ARMS-PCR/targeted NGS Real-time allele-specific PCR	Dabrafenib + trametinib Vemurafenib + cobimetinib
			cKIT mutations	Targeted NGS	Imatinib (not registered in this indication)
	CKAE1AE3	Undifferentiated carcinoma	EGFR, KRAS, BRAF, HER2, ALK, ROS1, RET, c-MET	ARMS-PCR/targeted NGS Real-time allele-specific PCR, IHC, FISH or RNA seq	
Adenocarcinoma (glandular ± mucosecretion)	Mucins, TTF1, p40, CK7, CK20	Unknown primary TTF1−, CK 7+/−, CK20−			
		Broncopulmonary adenocarcinoma (NSCLC) TTF1+, CK 7+, CK20−	EGFR-activating mutations	Targeted NGS or real-time allele-specific PCR (FDA-approved COBAS© EGFR mutation test v2, ARMS-PCR)	Gefitinib, erlotinib, afatinib
			EGFR resistant mutation: T790M	COBAS© EGFR mutation test v2 (tumor tissue and plasma) Dropet digital PCR (plasma), targeted NGS	Osimertinib
			ALK translocation	IHC, FISH, or RNA seq (for IHC+)	Crizotinib
			ROS1 translocation	IHC, FISH, or RNA seq (for IHC+)	Crizotinib, ceritinib, brigatinib, lorlatinib (not registered)
			RET translocation	FISH or RNA seq	Cabozantinib, vandetanib (not registered in this indication)
			c-MET amplification	FISH or targeted NGS	Crizotinib (not registered in this indication)
			c-MET splice-mutation (exon 14 skip mutation)	Targeted NGS	Crizotinib
			BRAF V600E or V600K mutations	Targeted NGS Real-time allele-specific PCR IHC (V600E)	Dabrafenib + trametinib Vemurafenib + cobimetinib
		Breast adenocarcinoma TTF1−, RE/RP+/− (GATA3+, mammaglobin+), CK 7+, CK20− (triple negative: CK5/6/14+)	HER2 amplifications	FISH, IHC	Trastuzumab, pertuzumab, lapatinib, trastuzumab-emtansine (TDM1)
		Colorectal adenocarcinoma CK 7−, CK20+, CDX2+	KRAS, NRAS mutations	ARMS-PCR/targeted NGS Allele-specific PCR-COBAS© KRAS mutation test	EGFR mAb (cetuximab, panitumumab) resistance
		Renal adenocarcinoma CK 7−, CK20−, vim+, CD10+			
		Rare cancers PSA, CA125, TG, PLAP, alpha-fetoprotein, β HCG			
Squamous cell carcinoma		Keratinization, intercellular bridges, p40+, CK5/6+, TTF1	FGFR1 amplification	FISH	Not registered drugs
			DDR2 mutations	Targeted NGS	Dasatinib (not registered in this indication)
Neuroendocrine carcinoma	CK+, TTF1+/−, chromogranin+, synapthophysin+	Small cell lung carcinoma (SCLC)	TTF1+		Not registered drugs
		Large cell neuroendocrine tumor	TTF1+/−		

*CK, cytokeratins; TTF1, thyroid transcription factor; IHC, immunohistochemistry; ARMS-PCR, amplification refractory mutation system-polymerase chain reaction; FISH, fluorescent in situ hybridization, NGS, next-generation sequencing; RNA seq, RNA sequencing; EGFR, epidermal growth factor; KRAS, Kirsten rat sarcoma viral oncogene homolog; BRAF, v-raf murine sarcoma viral oncogene homolog B; NRAS, rat sarcoma oncogene; HER2, human epidermal growth factor receptor 2; ALK, anaplastic lymphoma kinase; ROS1, repressor of silencing 1; RET, rearranged during transfection; c-MET, tyrosine-protein kinase Met or hepatocyte growth factor receptor; cKIT, tyrosine-protein kinase Kit or CD117; FGFR1, fibroblast growth factor receptor 1; DDR2, discoidin domain-containing receptor 2*.

With regard to EGFR status, lung adenocarcinomas carrying EGFR mutations have a higher risk of developing BM (64%), probably as a consequence of the prolonged survival due to treatment with EGFR-tyrosine kinase inhibitors (TKIs) (63, 64). In BM, EGFR status was evaluated in few studies. Porta et al. evaluated EGFR mutation status in 69 NSCLC-BM patients treated with erlotinib: 17 had EGFR mutation with an intracranial response rate of 82.4% (65, 66). ALK rearrangements occur in 3–7% of patients with NSCLC, but no data are available about the incidence in BM (67). Approximately 1.4% of NSCLCs harbor ROS1 rearrangements. ROS is strongly homologous to ALK, so that ALK inhibitors can be efficacious against ROS1-positive tumors (68). In NSCLC, multiple mechanisms of c-MET activation have been reported, including gene amplifications and exon 14 skip mutations (69).

In melanoma, BRAF mutations (mainly BRAF V600E) are found in 50% of melanoma BM, leading to a constitutive activation of the MAPK signaling pathway, promoting cellular growth, invasion, and metastasis (70).

The identification of BRAF mutations is relevant for therapeutic management, but it is not a diagnostic tool, given the fact that it is observed in numerous CNS tumors, notably in 10% of glioblastomas (in 50% of epithelioid glioblastomas), in over 60% of gangliogliomas and pleiomorphic xanthoastrocytomas, in 5% of BM of colon cancers, and in 1–4% of BM of NSCLC (71).

In breast cancer, 62–75% of patients maintain the same receptor status between the primary tumor and the brain metastasis, whereas discordance rates of 25–37.5% have been found. The rate of ER and PgR expression was 41.6% in the primary tumors and decreased to 12.5 and 16.6% in the BMs. By contrast, the rate for Her2+ tumors increased from 41.6% in primary breast cancer to 65.2%, respectively, in the BMs. All anti-estrogen treated breast tumors lost the ER expression in the BMs, whereas a Her2/neu conversion did not occur after treatment with trastuzumab. Therefore, the receptor status of the primary tumor is invalid for planning a targeted therapy against BMs, especially after hormone therapy. In these cases, new tissue collection by biopsy or resection should be required for more accurate decision-making (72).

## Biomarkers and Liquid Biopsy

The diagnosis and management of BMs still rely on histopathology and neuroimaging. However, considering tumor complex genetic profiles, more sophisticated methods of tumor analysis are under investigations in fluids (so-called liquid biopsy). Circulating biomarkers, including tumor nucleic acids, tumor cells (CTCs), and extracellular vesicles, which contain tumor DNA as well as other macromolecules, such as microRNA, have shown promise to provide information regarding tumor evolution over time, specifically for monitoring treatment response and disease progression (73, 74).

Although the technical aspects of biomarker detection still require optimization, these tools have already demonstrated their diagnostic, prognostic, and predictive value in several tumor types, including breast, CRC, non-small cell lung, prostate cancers, and melanoma. In addition, they can be used in the laboratory to probe mechanisms of acquired drug resistance and tumor invasion and dissemination (75, 76).

Genomic alterations can be detected in the CSF by micro-arrays, digital or real-time polymerase chain reaction (qPCR) and targeted amplicon sequencing, and whole exome sequencing (77, 78). Although a lumbar puncture is a more invasive procedure than a blood draw, the possible lack of representative tumor DNA in the plasma may lead to inconclusive results. CSF likely represents a preferable source of liquid biopsy in brain metastatic lesions featuring meningeal carcinomatosis, at least when no extra-CNS localizations are evident (79). It remains unclear in patients with metastatic solid tumors diagnosed with leptomeningeal carcinomatosis whether tumor DNA detection in the CSF compartment always reflects the local presence of cells or whether this DNA may be derived from tumor cells circulating in the blood or even from distant extracerebral metastases (80–82). Yang and colleagues have evaluated EGFR gene status in tumor-derived free DNA in CSF to test correspondence with the molecular pattern of the primary tumor and to guide the clinical use of EGFR-TKI. Indeed, between the paired primary tumor and tumor-derived DNA in CSF, 75% of samples had a concordant EGFR status (83). Findings on two patients by Marchiò and colleagues corroborate the notion that CSF represents a preferable source for liquid biopsy in brain metastatic lesions featuring meningeal carcinomatosis, at least when no extra-CNS localizations are evident (72). Liquoral liquid biopsy can allow the identification of either actionable genetic alterations or a mutation correlated to resistance to targeted therapies leading to crucial changes in the treatment decision.

## Therapy

The choice of therapeutic approaches, both in clinical trials and daily practice, is guided by the knowledge of the natural prognostic factors. Karnofsky performance status (KPS), age, primary/systemic tumor activity, neurocognitive function, number of BMs, primary tumor type, and time from primary tumor diagnosis to the brain lesion all have shown an individual prognostic significance in patients with BMs (84, 85). Of these, the KPS is the major determinant of survival. On the basis of most powerful factors, prognostic indices have been developed to distinguish subgroups of patients with different outcomes. The first prognostic categorization [Recursive Partitioning Analysis (RPA)], developed in 1997 by the Radiation Therapy Oncology Group (RTOG) (84), subdivided patients treated with whole-brain radiotherapy (WBRT) into three classes: class I including patients with a KPS greater than 70, controlled primary tumor/number of extracranial metastases and age below 65 years (median survival 7.7 months); class III including patients with KPS less than 70 (median survival 2.3 months); and class II including the rest of patients (median survival 4.5 months).

A new prognostic index, the diagnostic-specific Graded Prognostic Assessment (DS-GPA), derived from an updated RTOG database, has been proposed more recently, considered as prognostic as the RPA, less subjective and more quantitative (86). A further analysis has shown that the prognostic factors vary according to the tumor type (87). The DS-GPA uses four factors (age, KPS, status of extracranial disease, and number of BMs) to subdivide patients into one of four categories with median survivals ranging from 2.6 to 11 months. A new updated GPA, including molecular markers, has been proposed to better estimate survival in patients with BM: lung-mol-GPA is an update of the DS-GPA, which incorporates EGFR and/or ALK mutation status in addition to the well-known prognostic factors such as KPS, age, presence of extracranial metastases, and number of BMs (88). For melanoma there are five significant prognostic factors: age, KPS, extracranial metastases, number of BMs, and BRAF status (89). Conversely, significant prognostic factors for breast cancer are KPS, age and tumor subtype (classified HER2, ER, and PgR status) (90), but not the number of BMs and extracranial metastases.

## Surgery

Three randomized trials have compared surgical resection followed by WBRT with WBRT alone (91–93). The American and European trials have shown a survival advantage for patients receiving the combined treatment (median survival 9–10 vs 3–6 months). In the American study, patients who received surgery displayed a lower rate of local relapses (20 vs 52%) and a longer time of functional independence. The Canadian study, which included more patients with an active systemic disease and a low performance status, did not show any benefit with the addition of surgery to WBRT. Therefore, the survival benefit of surgical resection seems limited to the subgroup of patients with controlled systemic disease and good performance status. Surgical resection allows a relief of symptoms of intracranial hypertension, a reduction of neurological deficits and seizures, and a rapid steroid taper.

Gross total resection of a brain metastasis can be achieved with lower morbidity using advanced image-guided systems, such as preoperative functional MRI and DTI, intraoperative neuronavigation and cortical mapping (94, 95).

Leptomeningeal disease (LMD) can be a complication, especially for patients with posterior fossa metastases undergoing a “piecemeal” resection (13.8%) compared with “en-bloc” resection (5–6%) (96).

Surgery may be considered for patients with two to three surgically accessible BMs in good neurological condition and controlled systemic disease: a complete resection yields results comparable to those obtained in single lesions (97).

The use of carmustine wafers in the resection cavity in newly diagnosed brain metastasis has not been tested in prospective trials (98).

Another technique is the GliaSite Radiation Therapy System, an intracavitary high-activity 125-I brachytherapy, performed with a balloon placed in surgical cavity and filled with a radioactive solution, delivering highly localized doses of radiation to the resection margins (60 Gy to 1 cm depth). However, this technique has not been further developed after some interesting preliminary results (99).

## Stereotactic Radiosurgery (SRS)

Stereotactic radiosurgery is a single, high-dose radiation treatment with precise localization of a target of 3–3.5 cm of maximum diameter by using gamma-knife (multiple cobalt sources) or linear accelerator (Linac) through a stereotactic device. The steep dose fall-off minimizes the risk of damage to the normal nervous tissue. Most BMs are an ideal target for SRS, owing to the small size, spherical shape, and distinct radiographic and pathologic margins (100). The dose is inversely related to tumor diameter and volume (101). In general local tumor control decreases as the size of metastasis increases and the dose that could be given decreases. Following SRS for newly diagnosed BMs a decrease of symptoms and a local tumor control (shrinkage or arrest of growth) at 1 year of 80–90% and a median survival of 6–12 months have been reported (102). Metastases from radioresistant tumors, such as melanoma and renal cell carcinoma, respond to SRS as well as do metastases from radiosensitive tumors (103). Radiosurgery allows the treatment of BMs in almost any location, including brainstem (104). The type of radiosurgical procedure, gamma-knife or Linac-based, does not have an impact on the outcome. SRS combined with WBRT (radiosurgical boost) is superior to WBRT alone in terms of survival (105) in patients with single lesions only. Survival following radiosurgery is comparable to that achieved with surgery (106). SRS is less invasive and can be performed in an outpatient setting, and offers cost effectiveness advantages over surgery; on the other hand, patients with larger lesions may require chronic steroid administration.

Acute (early) and chronic (late) complications following radiosurgery are reported in 10–40% of patients (107). Acute reactions (due to edema) occur more often within 2 weeks of treatment, and include headache, nausea and vomiting, seizures, and worsening of preexistent neurological deficits. These reactions are generally reversible with corticosteroids. Chronic complications consist of hemorrhages and radionecrosis (1–17%). Radiographically, a transient increase in the size of the irradiated lesion, with increasing edema and mass effect, with or without radionecrosis, cannot be distinguished from a tumor progression: MR spectroscopy and perfusion and PET techniques can give additional information (108, 109).

Hypofractionated radiosurgery is used for larger metastases (two to five fractions of smaller doses) to decrease the risk of radionecrosis and other neurological complications (110–112).

Most studies comparing SRS with surgical resection have reported similar outcomes in terms of survival for patients with oligometastases (113, 114). However, these are retrospective studies and are likely to be affected by selection bias.

Overall, the choice between surgery and SRS should be made considering many factors, such as number, location and size of BMs, neurologic symptoms, patient preference and physician expertise.

## WBRT Following Surgery or Radiosurgery

The rationale of WBRT in conjunction with surgery or SRS is that of destroying microscopic disease at the original tumor site or at distant intracranial locations: however, it has been questioned by recent trials (115). Three phase III trials (116–118) in patients with single brain metastasis have reported that the addition of WBRT to either surgery or SRS reduces the risk of local and distant relapses in the brain but does not improve overall survival. The American (116) and the Japanese (117) trials included patients with progressive systemic disease, while the EORTC 22952-26001 trial recruited patients with stable systemic disease, i.e., only those who could maximally benefit from the addition of early WBRT (118).

An individual patient data meta-analysis of three randomized trials assessing SRS with or without WBRT has challenged our current understanding of the effect of adding WBRT (119). The investigators reported a survival advantage for SRS alone in those patients presenting with one to four metastases, KPS of 70 or higher and age of 50 years or younger. Moreover, in the subgroup of patients with <50 years, a reduction in the risk of new BMs with adjuvant WBRT was not noted, while, in older patients (aged >50 years), WBRT decreased the risk of new BMs, but did not affect survival.

A secondary analysis of the Japanese trial has stratified patients by GPA score and suggested that a subgroup of patients with NSCLC with higher GPA scores (2.5–4.0) have a survival benefit from SRS + WBRT compared with SRS alone (median survival 16.7 vs 10.7 months) (120). However, a similar secondary analysis of the EORTC trial has not confirmed this finding (121).

The impact of adjuvant WBRT on cognitive functions and quality of life (QoL) has been analyzed in few studies (Table 3). Aoyama et al. compared, by using the Mini-Mental State Evaluation, the neurocognitive function of patients who underwent SRS alone or SRS + WBRT and showed a deterioration of neurocognitive function in long-term survivors (up to 36 months) after WBRT (120). Chang et al. in a small randomized trial have shown that patients treated with SRS plus WBRT were at greater risk of a decline in learning and memory function at 4 months after treatment compared with those receiving SRS alone (122).

**Table 3:** Phase III trials of SRS ± whole brain radiotherapy for brain metastases (BMs) with cognitive endpoints.

Reference	Inclusion criteria	Number of Pts	Primary endpoint	OS (months)	Outcome	Distant brain metastases and secondary endpoint
Chang et al. (122) (MDACC)	1–3 BMs	58	5-point drop on HVLT-R total recall at 4 months	SRS + WBRT: 5.7 SRS alone: 15.2 (*p* = 0.003)	Neurocognitive decline at 4 months: SRS + WBRT 52% SRS alone: 24%	SRS + WBRT: 73% SRS alone: 45% (*p* = 0.02) (at 1 year)
Brown et al. (287) (Alliance)	1–3 BMs	213	>1 SD in any of the six cognitive tests at 3 months	SRS + WBRT: 7.5 SRS alone: 10.7 (*p* = NS)	Neurocognitive decline at 4 months: SRS + WBRT 88% (*p* = 0.02) SRS alone: 62%	SRS + WBRT: 85% SRS alone: 51% (*p* < 0.01) (at 1 year)
Brown et al. (123)	Pts with 1–3 BM	213: – SRS alone, *n* = 111 – SRS plus WBRT, *n* = 102	Primary endpoint: cognitive deterioration (decline >1 SD from baseline on at least 1 cognitive test at 3 months) Secondary endpoint: time to intracranial failure, QoL, FI, long-term cognitive status, OS	m OS: – 10.4 ms for SRS alone – 7.4 months for SRS + WBRT (*p* = 0.92)	Cognitive deterioration at 3 months: – After SRS alone: 40/63 Pts (63.5%) – SRS + WBRT: 44/48 Pts (91.7%) Less deterioration with SRS alone (*p* < 0.001)	– Time to intracranial failure shorter for SRS alone compared with SRS + WBRT (*p* < 0.001) – For long-term survivors, cognitive deterioration was less after SRS alone at 3 months (45.5 vs 94%, *p* = 0.07) and at 12 months (60 vs 94%; *p* = 0.04) – QoL was higher at 3 months with SRS alone
Brown et al. (126) (NCCTG N107C/CEC·3)	1 resected BM and a resection cavity <5 cm	194 – SRS alone 98 Pts – WBRT 96 Pts	Cognitive-deterioration-free survival and OS	SRS: 12.3 months WBRT: 11.6 months	Median cognitive-deterioration-free survival: – SRS: 3.7 months – WBRT: 3 months – Cognitive deterioration at 6 months: SRS: 52% (28/54 Pts); WBRT: 85% (41/48 Pts)	

*MDACC, M.D. Anderson Cancer Center; Alliance, alliance for clinical trials in Oncology; Pts, patients; HVLT-R, Hopkins verbal learning test-Recall; SRS, stereotactic radiosurgery; WBRT, whole-brain radiotherapy; NS, not significant; OS, overall survival; BMs, brain metastases; QoL, quality of life; FI, functional independence*.

A randomized phase III trial (Alliance trial) has compared SRS alone vs SRS + WBRT in patients with one to three BMs using a primary neurocognitive endpoint, defined as decline from baseline in any six cognitive tests at 3 months. The decline was significantly more frequent after SRS + WBRT vs SRS alone (88 vs 61.9%) (class I) with more deterioration in immediate recall (31 vs 8%), delayed recall (51 vs 20%), and verbal fluency (19 vs 2%) (123).

A QoL analysis of the European Organisation for Research and Treatment of Cancer (EORTC) 22952-26001 trial has shown over 1 year of follow-up that patients receiving adjuvant WBRT had transient lower physical functioning and cognitive functioning scores and more fatigue (124). Based on the results of these trials, the American Society for Radiation Oncology (ASTRO) has recommended in its Choose Wisely campaign not to routinely add adjuvant WBRT to SRS for patients with a limited number of BMs, and the same has been done in the European Association of Neuro-Oncology guidelines (25). Importantly, in case of omission of WBRT, following either SRS or surgery, close monitoring with MRI is mandatory.

## SRS Following Surgery

Surgery alone in brain metastasis is associated with a high risk of local recurrence (118). Postoperative SRS is an approach to decrease local relapse and avoid the cognitive sequelae of WBRT. SRS has other advantages over WBRT, such as reduced number of fractions and a shorter break for chemotherapy during radiation, thus reducing the risk of systemic recurrences. Based on retrospective cohort studies, the results of a meta-analysis by Lamba and colleagues (125), suggest that SRS of the resection cavity may offer comparable survival and similar local and distant control as adjuvant WBRT. A phase III randomized trial has been conducted to evaluate postoperatively SRS vs WBRT in brain metastasis patients (126). The co-primary endpoints were cognitive-deterioration-free survival and overall survival; secondary endpoints were QoL, adverse events, and functional independence. This trial concluded that SRS to the surgical cavity results in improved cognitive outcomes, better preservation of QoL and functional independence compared with WBRT. Despite worse intracranial control, there was no difference in overall survival. In conclusion, after resection of a brain metastasis, SRS should be considered one of the standard of care, as a less toxic alternative to WBRT (123, 127, 128).

However, postoperative SRS can be associated with a higher risk of developing leptomeningeal disease (LMD). Some recent studies have reported an incidence of LMD in patients receiving SRS to the resection cavity around 12–14% and breast cancer patients can have a particularly high rate of LMD (129–131). Several questions remain regarding SRS after surgery, such as major risk of radionecrosis, the optimal dose and fractionation schedule, especially for large metastases (>3 cm). The risk of radionecrosis following postoperative SRS is higher (between 9 and 15%) than that reported in an EORTC study with WBRT following surgery or SRS alone (2.6%) and could be reduced by modifying the treatment schedule with multifraction SRS (132). The actual incidence over time is 5.2% at 6 months, 17.2% at 12 months and 34.0% at 24 months (133). Preoperative SRS could be an alternative to reduce the risk of radionecrosis (134).

Overall, the true incidence of biopsy proven radionecrosis is unknown, as many studies have reported a combination of pathologically proven and MRI suspected radionecrosis. Advanced neuroimaging techniques (MRI perfusion, MR spectroscopy, and PET with amino acids) may help diagnosis (109, 135). Treatment options for radionecrosis include corticosteroids and bevacizumab that could reduce edema and symptoms (136).

There are still few data regarding the impact of postoperative SRS on health related quality of life. Caveats of available studies comparing postoperative SRS and WBRT for BMs include the heterogeneity regarding primary tumor histology (with varying proportion of melanoma, colon, renal, breast, and unknown histologies), numbers of BMs and treatment modalities, which were not always clearly reported (125). Some studies described outcomes of intraoperative radiotherapy instead of SRS (137).

## WBRT Alone

Whole-brain radiotherapy alone is now the treatment of choice for patients with single or multiple BMs not amenable to surgery or radiosurgery, or, more in general, patients with poor prognostic factors and limited life expectancy. Tumor volume reduction after WBRT seems to be associated with better neurocognitive function preservation and prolonged survival (138). Median survival following WBRT alone ranges from 3 to 6 months, with 10–15% of patients alive at 1 year. A meta-analysis of 39 trials, involving 10.835 patients, concluded that altered WBRT dose fractionation schemes are not superior in terms of overall survival, neurologic function or symptom control as compared with standard fractionation (30 Gy in 10 fractions or 20 Gy in 5 fractions) (139). Currently, radiosensitizers, such as motexafin gadolinium (Gad) or efaproxiral, have not provided any additional benefit over conventional treatment in BMs from NSCLC or breast cancer (140, 141). Recently a phase III randomized trial (QUARTZ) randomly assigned 538 NSCLC patients with BMs, unsuitable for surgical resection or stereotactic radiotherapy, either to optimal supportive care (OSC) plus WBRT (20 Gy in five daily fractions) or OSC alone. The primary outcome measure was quality-adjusted life-years (QALYs): QALYs was generated combining overall survival and patients’ weekly completion of the EQ-5D questionnaire. An absence of a difference in QALYs and overall survival between the two groups was reported, suggesting that WBRT provides little additional benefit, over supportive care (i.e., dexamethasone), for this patient group (142).

The cognitive decline following WBRT has been linked to leukoencephalopathy, whose incidence is higher after WBRT vs SRS (143, 144). A study, evaluating 59 patients treated with primary SRS, found that even after multiple courses of SRS, QoL, measured with the EQ-5D instrument, was preserved in 77% of patients at 12 month follow-up (145). Thus SRS seems to be a valid treatment option that can help maintain neurocognition and QOL.

## Brain Damage Following WBRT

Radiation-induced brain injury can be categorized as acute, early-delayed or late-delayed reactions (146). Acute injury is very rare and occurs hours to weeks after radiation therapy and consists of somnolence, drowsiness, and headache. Early delayed injury occurs from 1 to 6 months post-irradiation and is characterized by drowsiness and transient demyelination. Both acute and early-delayed reactions are normally reversible with corticosteroids. Late delayed effects, characterized by demyelination, vascular abnormalities and ultimately white matter necrosis, are generally observed with a latency of 6 months or more post-irradiation, and are irreversible and progressive (147). Giving the prolonged survival of many cancer patients, long-term complications of WBRT could negatively impact the QoL of survivors. Currently, no treatments to prevent or reverse these effects are available.

Radiation-induced dementia with ataxia and urinary incontinence developed in up to 30% of patients by 1 year after receiving unconventional large size fractions of WBRT (6–8.5 Gy) (148). A diffuse hyperintensity of the periventricular white matter on T2-weighted and FLAIR images with associated hydrocephalus was observed on MRI. When using more conventional size fractions (up to 3 or 4 Gy per fraction) the risk is that of milder cognitive dysfunction, consisting of learning and memory impairment with changes in the white matter and cortical atrophy on MRI. Patients with advanced age and vascular disease (such as arterial hypertension) are at higher risk of developing cognitive dysfunction. The pathogenesis of this radiation-damage consists of an injury of the endothelium of microvessels, which leads to atherosclerosis and chronic ischemia: the clinical–radiological pictures are similar to those of small vessel disease.

To reduce the neurocognitive sequelae of WBRT pharmacological approaches have been investigated. The RTOG 0614 was a phase III trial investigating memantine, an *N*-methyl-d-aspartate receptor blocker, shown to be effective in vascular dementia, and included 508 patients with BM who were randomized to either WBRT plus placebo vs WBRT plus daily memantine for 6 months (149). Memantine has the potential to block excessive NMDA stimulation following ischemia, which causes damage of the normal brain. The study narrowly missed statistical significance for the primary endpoint of a lesser decline in delayed recall at 6 months. However, the memantine arm showed a longer time to cognitive decline, with probability of cognitive decline at 6 months being 54% in the memantine arm and 65% in the placebo arm. Other treatments, including donepezil and cognitive rehabilitation, have been tested in the population at risk for dementia, although they have not been adequately studied in patients with BM, following cranial radiotherapy (150). An innovative approach could be the use of intranasal metabolic stimulants, such as low dose insulin, which could be valuable in improving cognition and memory, by reversing impaired brain metabolic activity (151).

Radiation-induced cognitive deficits may result, at least in part, from a radiation injury to the neuronal stem cells in the subgranular zone of the hippocampus (152, 153), that are responsible for maintaining neurogenesis, and preserving memory functions. Preclinical and clinical studies have shown that radiation-induced injury to the hippocampus correlates with neurocognitive decline following WBRT. Hippocampal sparing WBRT uses intensity modulated radiotherapy (IMRT) to conformally reduce the radiation dose to the hippocampus, while applying the usual higher dose to the whole brain (154). A potential concern is whether hippocampal avoidance could lead to loss of control of metastases in the limbic structures: however, the hippocampus does not seem to be frequently involved in the metastatic process (155, 156). The single-arm phase II RTOG 0933 study has suggested that conformal avoidance of the hippocampus during WBRT is associated with some sparing of memory and QoL: performance on standardized memory tests declined 7% from baseline to 4 months in patients treated with HS-WBRT, as compared with 30% in historical control group (157).

The potential combined neuroprotective effects of hippocampal avoidance in addition to memantine during WBRT for BMs are being investigated in a phase III trial (158).

## Treatment of Recurrent Brain Metastasis

Treatment of recurrent BMs with salvage surgery or SRS is used in an increasing number of patients. Reoperation can afford a neurological improvement and prolongation of survival in patients with a local accessible brain lesion, high performance status, stable extracranial disease and relatively long time to recurrence (>6 months) (159, 160). Arbit et al. analyzed 109 patients with recurrent BMs of NSCLC. Thirty-two patients, who underwent surgery at relapse, had significantly longer median survival (15 months) than 77 patients in whom surgical intervention was omitted (10 months, *p* < 0.001) (161).

Salvage SRS after WBRT has been widely used during the initial development of SRS: several retrospective studies have reported reasonable local control and survival rates (162–165). A population-based study has suggested similar survival outcomes following either salvage SRS or boost SRS (166). Reirradiation with SRS after local recurrence of an initial SRS has been employed in a limited number of patients thus far, and the risk of long-term radionecrosis should be considered against the benefit (167). Multiple courses of SRS for new BMs after an initial course of SRS to defer WBRT could yield high rates of local control, low risk of toxicity, and favorable duration of overall and neurologic progression-free survival (PFS) (168, 169). When using multiple courses of SRS the aggregate volume, but not the cumulative number of BMs, and the GPA score, as recalculated at the second course of SRS, are seen as critical for survival (170). Salvage WBRT following previous WBRT or SRS is rarely employed.

## Chemotherapy and Targeted Therapies

Unfortunately, few clinical trials of systemic agents have been conducted to date in patients with BM, and this population has frequently been excluded from clinical trials of emerging investigational drugs (171). Historically, the use of systemic therapy in patients with BMs has been limited by the presence of the BBB, which limits the access of hydrophilic and/or large agents into the CNS. However, the BBB is disrupted in macroscopic BMs resulting in an increased exposure to systemic drugs. An additional increase in BBB permeability can be induced by radiotherapy, even if unpredictably. The sensitivity of tumor cells to cytotoxic chemotherapy appears to be as important as the BBB: response rates are high in BMs from small cell lung cancer (30–80%), intermediate in breast cancer (30–50%) and NSCLC (10–30%), and low in melanoma (10–15%), and responses in the brain do not always parallel that of the systemic disease (172, 173). Because active BMs often coexist with active systemic disease, antitumor agents that can control both intracranial and extracranial disease are needed.

The combination of radiotherapy and chemotherapy may improve response rate, but not survival (174). Various chemotherapeutic agents have been employed to treat BM, often used in a combination of two to three agents along with WBRT. Temozolomide alone has a modest therapeutic effect with some improvement when used in conjunction with WBRT and/or other anticancer agents (175).

Brain metastases differ from metastases to other organs from a biologic and clinical perspective. The brain has a unique micro environment and an immune system distinct from other organs (176). Other considerations, which could explain the disappointing results of chemotherapy in BM, are the fact that metastatic cells in the brain often develop after multiple rounds of prior chemotherapies for the systemic disease, allowing for the development of resistance through the accumulation of different mutations. Second, the breakdown of BBB is heterogeneous in BM, preventing an optimal drug distribution. Finally, BM patients may develop neurologic deficits and seizures, which could require the use of medications (i.e., enzyme-inducing anticonvulsants, corticosteroids), that accelerate the metabolism of antitumor agents (177).

Recent advances in understanding the molecular basis of tumor growth in many solid tumors have allowed the development of agents targeting molecular pathways both in the extracranial and intracranial disease (172, 178–180). Encouraging results have emerged for tyrosine kinase inhibitors and monoclonal antibodies in subgroups of patients with BM (Table 4). As CNS immune-accessibility has become accepted, and immunotherapy (IT) gains greater understanding within trials for primary brain tumors, there is an increasing interest in immunotherapeutic approaches to BMs with immune checkpoint inhibitors (181–183) (Table 5).

**Table 4:** Targeted agents in brain metastases (BMs).

Main studies on targeted therapies in BMs				Results
Reference	Agent tested	Number of Pts	Clinical trial characteristics	
**NSCLC**				
Sperduto et al. (199)	Erlotinib	126	Phase III multicenter trial (RTOG 0320) Three arms: arm 1: WBRT + SRS; arm 2: WBRT + SRS + TMZ; arm 3: WBRT + SRS + erlotinib EGFR mutation not tested	MST: arm 1 (44 Pts): 13.4 months; arm 2 (40 patients): 6.3 months; arm 3 (41 Pts): 6.1 months CNS mPFS: arm 1: 8.1 months; arm 2: 4.6 months; arm 3: 4.8 months
Welsh et al. (197)	Erlotinib	40	Phase II study of erlotinib plus WBRT Erlotinib started 1 week before WBRT	ORR 86% (*n* = 36) MST 11.8 months 9 of 17 patients tested for EGFR mutation were positive MST in EGFR mutant: 19.1 months MST in EGFR wild-type 9.3 months
Rosell et al. (288)	Erlotinib + bevacizumab	109	Phase II trial, single-arm, multicentre	Overall median PFS: 13.2 months in T790M-positive group Median PFS: 16.0 months in the T790M-positive group while it was 10.5 months in T790M-negative
Ceresoli et al. (289)	Gefitinib	41	Phase II single-arm study No concurrent local therapy for BM Both squamous and adenocarcinoma included EGFR mutation not tested	ODC 27% mPFS 3 months Disease control better in patients who received prior WBRT and who had adenocarcinoma histology
Costa et al. (205)	Crizotinib		Retrospective analysis of trials	Poor activity against CNS metastasis in NSCLC as evidenced by low concentrations detected in CNS samples
Gadgeel et al. (290)	Alectinib	50	Pooled analysis of two phase II studies 136 patients had BM, 50 had measurable disease ALK-positive NSCLC, previously treated with crizotinib	CNS ORR 64% CNS DOR 10.8 months
Shaw et al. (291)	Ceritinib	122	Phase I study in advanced ALK-positive NSCLC	The ORR was 58% Among the 80 patients who failed crizotinib, the response rate was 56% NSCLC who received ceritinib with doses 400 mg or higher, the mPFS was 7.0 months
Crinò et al. (292)	Ceritinib	140 (100 with BM)	Phase I	Intracranial overall response rate: 45.0% (95% CI, 23.1–68.5%) Ceritinib treatment provided clinically meaningful and durable responses with manageable tolerability in chemotherapy- and crizotinib-pretreated patients, including those with brain metastases
Gettinger et al. (293)	Brigatinib (dual inhibitor of ALK and EGFR)	137	Phase I/II single-arm, open-label, multicenter study in patient Pts with advanced NSCLC	50 Pts with BM Eight (53%) of 15 assessable patients with measurable BM had an intracranial response 11 (35%) of 31 assessable patients with only non-measurable BM had complete disappearance of lesions on imaging Median intracranial PFS for these Pts is 97 weeks
Yang et al. (294)	Osimertinib		Phase II AURA trial	Activity in patients with CNS metastases: 16 (64%) of 25 evaluable Pts had an objective response, including 4 complete responses Median PFS in Pts with CNS metastases was encouraging, although it was shorter than in those without (7.1 vs 13.7 months, respectively) Benefit was observed across all predefined subgroups, including patients with asymptomatic CNS metastases at baseline (PFS: 8.5 vs 4.2 months)
Planchard et al. (295)	Dabrafenib and trametinib	57 total (only one patient with asymptomatic BM)	Phase II, multicentre, non-randomized, open-label study	Dabrafenib plus trametinib is a promising new therapy for patients with BRAFV600E-mutant NSCLC, with high overall response, a prolonged duration of response, and manageable toxicity. Few data on efficacy on BM

**Breast cancer (HER2-positive)**				
Bachelot et al. (231)	Lapatinib + capecitabine	45	Single-arm phase II study No prior WBRT, or lapatinib or capecitabine	CNS ORR 65.9% CNS mPFS 5.5 months mOS 17 months
Lin et al. (230)	Lapatinib + capecitabine	50	Phase II trial	CNS response of 20%PFS not reported
Cortes et al. (296)	Afatinib	121	Phase II, multicenter, randomized trial, open-label, three-arm study Arm A: afatinib, arm B: afatinib +vinorelbine, arm C: investigator choice	Arm A: PB = 30% Arm B: PB = 34.2% Arm C: PB = 41.9%
Freedman et al. (232)	Neratinib	40	Multicenter, open-label phase II study Patients who had progressed after one or More lines of CNS directed therapy	CNS ORR: 8% mPFS 1.9 months mOS 8.7 months

**Melanoma**				
Long et al. (243)	Dabrafenib	172	Multicenter, open-label phase II study V600E or V600K BRAF-mutant patients Two cohorts: cohort A: BM treatment naive, cohort B: previously treated BM	Cohort A, V600E (74 patients) – CNS ORR 39.2% – CNS PFS 16.1 weeks – OS 33.1 weeks Cohort A, V600K (15 patients) – CNS ORR 6.7% – CNS PFS 8.1 weeks – OS 31.4 weeks Cohort B, V600E (65 patients) – CNS ORR 30.8% – CNS PFS 16.6 weeks – OS 16.3 weeks Cohort B, V600K (18 patients) – CNS ORR 22.2% – CNS PFS 15.9 weeks – OS 21.9 weeks
McArthur et al. (245)	Vemurafenib	146	Open-label, phase II study Two cohorts: – Cohort-1: previously untreated (90 Pts) – Cohort-2: previously treated (56 Pts)	Cohort-1: – CNS ORR 18% – CNS PFS 3.7 months – OS 8.9 months Cohort-2: – CNS ORR 23% – CNS PFS 4.0 months – OS 9.6 months

*WBRT, whole-brain radiation therapy; SRS, stereotactic radiation; TMZ, temozolomide; Pts, patients; mPFS, median progression-free survival; EGFR, epidermal growth factor receptor; MST, median survival times; CNS, central nervous system; ODC, overall disease control; ORR, overall response rate; DOR, duration of response; mOS, median overall survival; PB, patient benefit (defined as intracranial or extracranial progression-free survival, no new neurologic signs or symptoms related to tumor, increase corticosteroid use) at 12 weeks; BM brain metastases; CT, chemotherapy*.

**Table 5:** Immunotherapy (IT) in BMs.

Main studies on IT (checkpoint inhibitors) in BMs					Results
Reference	Agent tested	Primary tumor	Number of pts	Clinical trial characteristics	
Margolin et al. (244)	Ipilimumab	Melanoma BM	72	Single-agent, open-label phase II study	Cohort A: 51 asymptomatic Pts with active BM who were not on corticosteroids; cohort B: 21 Pts with symptomatic melanoma-derived BM taking a corticosteroid Immune-related response criteria: the RR of 25% (13 Pts) and 10% (2 Pts) in cohorts A and B, respectively mOS: 7.0 months in cohort A and 3.7 months in cohort B
Di Giacomo et al. (252)	Ipilimumab and fotemustine	Melanoma BM	20/83 with asymptomatic BM	Open-label, single-arm, phase II trial	The mPFS in Pts with BM was 3.0 months 3-year follow-up of group with BM: mOS of 12.7 months
Patel et al. (297)	SRS vs SRS + ipilimumab	Melanoma BM	SRS: 34 SRS + ipilimumab: 20	Restrospective comparative analysis	SRS: 1 year LC 91%; 12 months OS: 38% SRS + ipilimumab: 1 year LC 71%; 12 months OS 37%
Weber et al. (298)	Ipilimumab/budesonide (asymptomatic BM)	Melanoma BM	12	Retrospective analysis of phase II trial	Intracranial RR: 41.6% mOS: 14
Knisely et al. (261)	SRS vs SRS + ipilimumab	Melanoma BM	SRS: 17 SRS + ipilimumab: 11	Retrospective comparative analysis	SRS: OS 4 months SRS + ipilimumab: OS 21.3 months
Silk et al. (299)	SRS vs SRS + ipilimumab	Melanoma BM	SRS: 37 SRS + ipilimumab: 33	Retrospective comparative analysis	SRS: PFS 3.3 months; OS 5.3 months SRS + ipilimumab: PFS 2.7 months; OS 8.3 months
Mathew et al. (300)	SRS vs SRS + ipilimumab	Melanoma BM	SRS: 33 SRS + ipilimumab: 25	Retrospective comparative analysis	SRS: 6-month OS 56% SRS + ipilimumab: 6-month OS 45%
Ahmed et al. (265)	SRS/nivolumab	Melanoma BM	26	Retrospective analysis	OS 11.8 months
Goldberg et al. (221)	Pembrolizumab	NSCLC, melanoma BM	36 (18 NSCLC, 18 melanoma)	Phase II trial	Brain metastasis response was achieved in 4 (22%) of 18 Pts with melanoma and 6 (33%) of 34 Pts with NSCLC. Responses were durable
Schachter et al. (301)	Pembrolizumab	Melanoma BM	834		Median follow-up was 22.9 months Median overall survival was not reached in either pembrolizumab group and was 16 months with ipilimumab Pembrolizumab continued to provide superior overall survival vs ipilimumab
Parakh et al. (302)	Nivolumab and pembrolizumab	Melanoma BM	66	Retrospective analysis	The IC overall RR was 21% and disease control rate 56%. Responses occurred in 21% of Pts with symptomatic BM. The median OS was 9.9 months (95% CI 6.93–17.74). Pts with symptomatic BM had shorter PFS than those without symptoms (2.7 vs 7.4 months, *p* = 0.035) and numerically shorter OS (5.7 vs 13.0 months, *p* = 0.068). Pts requiring corticosteroids also had a numerically shorter PFS (3.2 vs 7.4 months, *p* = 0.081) and OS (4.8 vs 13.1 months, *p* = 0.039)

*Pts, patients; BMs, brain metastases; NSCLC, non-small-cell lung cancer; RR, response rate; mOS, median overall survival; mPFS, median progression-free survival; 1 year LC, local control; IC RR, intracranial response rate*.

Overall, the response rate to targeted agents in specific molecular subtypes of BMs seems now higher than those observed after conventional chemotherapy. However, even if the majority of targeted agents are small molecules, still the passage across the BBB is critical, as most of these new compounds, similar to the old chemotherapeutics, have been shown to be a substrate of one or more active efflux transporters (i.e., PgP signaling).

### BMs From NSCLC

The survival of the general metastatic NSCLC population is approximately 12 months, with a median PFS ranging from 3 to 6 months. BMs are associated with poor prognosis, and the median survival ranges from 2.4 to 4.8 months for patients who receive palliative radiotherapy.

Platinum compounds (cisplatin and carboplatin) and pemetrexed, alone or in association (etoposide, vinorelbine, and radiotherapy) are the most commonly used chemotherapeutics against BMs from NSCLC (184). Temozolomide has shown some activity.

In the last decades, a multitude of molecular abnormalities have been discovered in NSCLC.

Approximately 33% of patients with NSCLC tumors and EGFR-TKI-sensitizing mutations develop BM (185). A pooled analysis including 464 patients from 16 trials to study the efficacy of first generation EGFR-TKIs (gefitinib, erlotinib) in NSCLC patients with BM showed significant beneficial effects, with a higher response rate (85 vs 45.1%) for EGFR mutated vs wild-type tumors and a median PFS of 7.4 months, and OS of 11.9 months in the EGFR mutation group (186). These data suggest that EGFR-TKIs are an effective treatment for NSCLC patients with BMs harboring activating EGFR mutations. However, even in EGFR wild-type patients, EGFR-TKIs seem to represent a potential second-line therapy with a response rate of about 10% (187). Evidence suggests that first generation EGFR-TKIs have limited BBB penetration (188, 189).

Afatinib is a second-generation irreversible covalent inhibitor of the EGFR tyrosine kinase including ErbB-2 (HER2) and ErbB-4. Subgroup analysis from LUX-LUNG 3 and LUX-LUNG 6 studies demonstrated a significant overall survival benefit for afatinib compared with chemotherapy in stage IIIB/IV lung adenocarcinoma patients with 19del-EGFR mutation (190). In a combined *post hoc* analysis of both studies, PFS was significantly improved with afatinib vs chemotherapy in patients with BM (8.2 vs 5.4 months; *p* = 0.0297) (191). Afatinib has reported good results in some cases of LM in stage IV exon 19-del-EGFR-mutant lung adenocarcinoma in association with WBRT, resulting in an almost complete regression of neurological symptoms as well as good, durable radiological responses (192). A good brain response in a patient with EGFR-mutant lung adenocarcinoma and multiple BMs who switched from erlotinib to afatinib due to hepatotoxicity, has been reported (193).

Based on the high intracranial response rates, TKIs have been hypothesized to be used alone as initial treatment in patients harboring activating EGFR mutations and asymptomatic small BMs (64, 194, 195). The main advantages of using TKIs alone are that patients can potentially avoid the adverse effects of WBRT as long as the intracranial disease is well controlled. The disadvantages could be that the discordance rate of EGFR mutations between the primary tumor and BMs can be as high as 32%, and the CSF penetration rate of gefitinib (1–10%) and erlotinib (2.5–13%) is limited. A meta-analysis has suggested that cranial radiotherapy (SRS or WBRT) associated with TKIs is more effective in improving response rate and disease control rate than radiotherapy alone or chemotherapy. Moreover, radiotherapy plus EGFR-TKIs significantly prolonged the median overall survival but significantly increased adverse events of any grade, especially rash and dry skin (196).

On the other hand, clinical trials offering a combination of erlotinib with radiation therapy (SRS or WBRT), in patient cohorts not specifically selected for target expression (197–199), has failed to demonstrate a superiority over radiotherapy alone. The use of up-front icotinib was recently evaluated in untreated patients in comparison with first-line chemotherapy (cisplatin plus pemetrexed) (200) or WBRT followed by chemotherapy (201). PFS was significantly longer for icotinib in both trials: PFS of 11.2 months in the icotinib group vs 7.9 in the chemotherapy group, an intracranial PFS of 10 months with icotinib vs 4.8 months with WBRT and CT.

Osimertinib, a third-generation EGFR-TKI, that targets activating mutations (EGFRm) and resistance mutations (T790M), has demonstrated robust systemic activity and a better CNS penetration with sustained tumor regression of BM. In the phase I BLOOM study, two third-generation EGFR-TKIs, osimertinib and AZD3759, were studied in patients with EGFR mutation-positive advanced NSCLC with BM and LM, showing improved BBB penetration and preliminary interesting results (202, 203).

Other “druggable” alterations seen in up to 5% of NSCLC patients are the rearrangements of the ALK gene. In particular, ALK translocations have been found in 3% of BMs from NSCLC and seem to be maintained in brain metastasis (204). NSCLC with ALK activating translocations are sensitive to the ALK inhibitor crizotinib. A recent study on BMs from ALK-rearranged NSCLC (205) has reported that crizotinib was associated with more than 55% disease control within the CNS at 3 months of therapy in both RT-naïve and RT-pretreated patients; moreover, crizotinib was associated with a moderate (18–33%) RECIST-confirmed response rate. However, the CNS is a common site of progression in NSCLC receiving crizotinib (206). Crizotinib, in addition of blocking ALK and ROS1, is also a potent c-MET inhibitor: clinical trials using crizotinib are ongoing in patients with NSCLC and c-MET mutations (207).

The US Food and Drug Administration approval of ceritinib and alectinib (second-generation of ALK inhibitors) for patients failing crizotinib means that ALK TKIs with improved CNS penetration are now available. The recently reported ASCEND-4 study (208) compared ceritinib with chemotherapy for treatment-naive patients with advanced ALK-positive NSCLC; this trial allowed both untreated as well as symptomatic BM to be accrued. Ceritinib achieved superior PFS and RR, with a similar trend seen in the subset of patients with BM. In the 22 patients with measurable CNS disease, ceritinib achieved a 73% CNS RR, with 86% of patients without CNS progression at 24 weeks. The J-ALEX study compared alectinib with crizotinib in patients with TKI-naive advanced ALK-positive NSCLC and demonstrated an overall PFS improvement: while only 21% of the patients had BM, the PFS benefit in these patients was dramatic (hazard ratio, 0.08) (209).

Third-generation ALK inhibitors, such as brigatinib and lorlatinib (a selective brain-penetrant ALK/ROS1TKI active against most known resistance mutations) have shown efficacy on BM in initial studies (210–212).

Another compound investigated in association with WBRT in BMs from breast and NSCLC is veliparib, a PARP 1–2 inhibitor. Despite preliminary encouraging safety and efficacy results (213); a phase II, randomized study evaluating the efficacy and safety of veliparib in combination with WBRT vs WBRT plus placebo in patients with BMs from NSCLC did not show significant differences in OS, intracranial response rate, and time to progression (214).

c-MET is an oncogenic driver and is implicated in the resistance to targeted therapies, including EGFR and VEGFR inhibitors. Increased c-MET expression may predict response to c-MET-targeted drugs (215, 216). Since c-MET amplification can contribute to acquired resistance to EGFR-TKI therapy, combined inhibition of EGFR and c-MET is being investigated (217).

Nivolumab, a checkpoint inhibitor, is a human IgG4 anti-PD-1 monoclonal antibody active in the second-line treatment of metastatic NSCLC after progression on a platinum-based chemotherapy (218). Intracranial activity and responses to nivolumab in a small cohort of five patients with NSCLC and untreated/progressive BMs suggested a role for this molecule in brain disease control. Importantly, no grade 3/4 adverse events were seen. Systemic responses and intracranial responses were largely concordant. However, no firm conclusions can be drawn due to the small sample size of the cohort (219).

An analysis presented at American Society of Clinical Oncology (ASCO) 2016 pooled data of patients with advanced NSCLC and pretreated asymptomatic CNS metastases from CheckMate 063 (phase II), 017 (phase III), and 057 (phase III). The best response in the nivolumab arm with CNS metastases arm was CR/PR in 28%, SD in 33%, and PD in 39% compared with CR/PR in 19%, SD in 31%, and PD in 43% in the docetaxel arm. Among patients with pretreated CNS metastases (74% had prior radiotherapy and 85% had ≥2 extra-CNS sites of metastases), median OS was a little longer in the nivolumab group (8.4 months) compared with the docetaxel group (6.2 months). Nivolumab was well tolerated, with low-grade toxicities. One-third of patients had no evidence of CNS progression at time of PD/last assessment. Additional results (including OS and CNS progression rates in patients with/without pretreated CNS metastases and safety/efficacy of nivolumab in patients with untreated CNS metastases from CheckMate 012) will be presented shortly (220). Overall, these results support further investigation of nivolumab monotherapy in patients with NSCLC and asymptomatic CNS metastases.

Pembrolizumab, a fully human anti-PD-1 monoclonal antibody, is approved in first- and second-line treatment of metastatic NSCLC. Early data in NSCLC demonstrated that there is a response in BMs similar to that of the systemic disease (221). The median OS was 7.7 months to date. The available data for the use of anti-PD-1 agents in the treatment of BM do not yet include information on PD-L1 status.

### BMs From SCLC

Various combinations of etoposide, teniposide, cisplatinum, or carboplatinum are active against BMs (173). So far, no targeted agents are available.

### BMs From Breast Cancer

Chemotherapy regimens that combine cyclophosphamide, 5-FU, methotrexate, vincristine, cisplatin, and etoposide are active in patients with BMs from breast cancer (173). Capecitabine, belonging to the class of fluoropyrimidines, is an active drug (222). Likewise, high-dose methotrexate is effective in recurrent BMs (223), but a risk of leukoencephalopathy exists when administered after WBRT.

In HER2-positive, breast cancer targeted therapies have been widely used. Trastuzumab, which crosses a disrupted BBB within established BMs, could be active (224). Several case reports and small patients’ series indicate an activity of the antibody-drug conjugate T-DM1 (trastuzumab-emtansine) (225, 226). Few data are available on the combination of different anti-HER2 agents. The use of pertuzumab, a monoclonal antibody, that binds HER2 on another epitope than trastuzumab, in combination with trastuzumab and docetaxel, leads to a substantial improvement in progression-free and overall survival and may delay CNS disease onset (227, 228).

The dual EGFR and HER2 tyrosine kinase inhibitor lapatinib has shown moderate antitumor activity in HER2-positive BM (229). In a phase II study in HER 2+ breast cancer patients with BMs, following trastuzumab-based systemic chemotherapy and WBRT (230), CNS objective responses to lapatinib were observed in 6% of patients, and 21% experienced ≥20% volumetric reduction. A recent phase II single-arm study (LANDSCAPE) has shown that the association of lapatinib and capecitabine in patients with previously untreated BMs from HER-positive metastatic breast cancer yields durable responses in up to 65% of patients (231). Based on these results a randomized trial comparing lapatinib and capecitabine vs WBRT has been launched.

A single-arm phase II trial on neratinib, an irreversible TKI of HER 2, has shown a response rate of 8% with an OS of 8.7 months in patients with BMs, pretreated either with WBRT or SRS (232). Despite the advances in treating HER2-positive breast cancer, many questions remain unanswered, such as how to impact prior resistance or affect a sanctuary site, and the optimal use of these novel compounds with regard to disease setting, treatment sequence, and combination regimens (233).

There are no available data on the efficacy on BM of endocrine therapies. Another druggable pathway in breast BM is the phosphatidylinositol 3 kinase/Akt/mammalian target of rapamycin (mTOR) pathway, which is dysregulated in a significant number of HR-positive breast cancers (234).

Everolimus, an mTOR inhibitor, approved for the management of HR-positive, postmenopausal breast cancer patients, in combination with an aromatase inhibitor (235) has also shown CNS penetration with activity against subependymal giant-cell astrocytomas associated with tuberous sclerosis (236), and ongoing studies are testing the activity of everolimus in BM. Other small-molecule inhibitors, like abemaciclib (CDK 4/6 inhibitor), are being evaluated in breast cancer BM (237). PARP inhibitors are being investigated in BMs from triple negative breast cancer.

### BMs From Melanoma

Approximately half of advanced melanoma patients will develop BM (MBMs). The median OS for patients with MBMs is 4–5 months (87, 238).

Fotemustine (response rate of 5–25%) and temozolomide (response rate 6–10%), either as single agents or in combination with WBRT, are the most active chemotherapeutics against BMs from melanoma (173, 239). Novel targeted and immunotherapeutic agents have revolutionized the systemic management of melanoma. A number of prospective clinical trials have demonstrated that these agents, either alone or in combination, can prolong PFS and OS (240–242). Several studies have assessed the impact of these agents in patients with MBMs in a prospective setting (243–245).

The immune checkpoint inhibitors ipilimumab [targeting the cytotoxic T-lymphocyte antigen 4 (CTLA-4)] and nivolumab have led to astonishing results and unexpected long-term survival gains in advanced/unresectable melanoma (246, 247). It is of relevance that both compounds interfere with T-cell signals (248).

Ipilimumab showed activity in melanoma BMs, particularly if asymptomatic, and improved OS (244, 249, 250). An open-label phase II multicenter US trial (244) has shown that ipilimumab has activity in those patients with melanoma BMs, who do not need corticosteroids: disease control (CR + PR + SD) after 12 weeks of treatment was 16% in the cohort of asymptomatic patients without corticosteroids compared with 5% in the cohort of symptomatic patients receiving corticosteroids. Importantly, the investigators did not report any neurological adverse event as an effect of an inflammatory response to treatment in the CNS, even in patients who received prior radiation therapy. The possibility remains that steroid treatment at initiation of ipilimumab could abrogate or downmodulate the immune response. One of the larger studies to investigate ipilimumab evaluated 127 patients and demonstrated an OS benefit (93 vs 42 weeks, *p* < 0.0028) for patients who received concomitant IT and RT (251).

A single-arm phase II trial of ipilimumab in combination with fotemustine in patients with melanoma and asymptomatic BMs showed intracranial disease control in 50% of patients and a median OS of 13.4 months (252). A phase III trial of this combination is currently ongoing. A triple-arm phase III clinical trial will compare the OS at 2 years of fotemustine monotherapy, ipilimumab and fotemustine, and ipilimumab and nivolumab in patients with metastatic melanoma with BMs. This study (NCT02460068) is currently recruiting participants and is not expected to reach completion until 2020.

The anti-PD-1 antibodies nivolumab and pembrolizumab have demonstrated highly durable response rates (41 and 38%, respectively) in large phase I trials (253, 254), that were confirmed in subsequent phase III trials (255) and in the second-line setting after failure of anti-CTLA-4 therapy (240, 256). These agents in combination with ipilimumab are currently investigated in several ongoing phase II trials in advanced melanoma patients with BM (257, 258).

Barker and colleagues reviewed the clinical outcomes of the combination of ipilimumab and RT in melanoma, including BM (259). Radiographically, it was noted that an increase of brain metastasis size >150% occurred in 40% of the tumors treated with SRS before or during ipilimumab, while this occurred in 10% of metastases treated with SRS after ipilimumab. Hemorrhage was also noted after SRS during ipilimumab in 42% of treated BMs. Preliminary results, that need further study, suggest an interaction between IT and RT. Also the reported “abscopal effect” in melanoma patients, in whom radiotherapy for one lesion induced a shrinkage of non-irradiated lesions, probably depending on the activation of an antitumor immune response, supports the potential of combining radiotherapy and IT in the treatment of melanoma (260).

Other several small, retrospective series have evaluated patients with melanoma BMs treated with IT and SRS reporting successful outcomes in terms of OS (261, 262). Qian et al. investigated melanoma BM patients treated concurrently (within 4 weeks of IT) with immune checkpoint inhibitors and SRS (defining “concurrent” when SRS was administered): the median percentage of the reduction in lesion volume was significantly greater for the concurrent group without hemorrhagic complications (263). In contrast to this report, the preliminary data reported by Shen et al. showed an increase in lesion size in 13 of 26 lesions treated concurrently (defined as IT starting “prior to or with SRS”) (264). A recent phase I prospective study explored the maximum tolerable dose and safety of ipilimumab with SRS or WBRT in patients with BMs from melanoma, demonstrating the safety of combining SRS with either ipilimumab 3 or 10 mg/kg.

The higher rate of increasing lesions as well as radionecrosis among patients receiving SRS or WBRT in combination with immune checkpoints inhibitors is a matter of debate, but many authors believe that these findings could be an expression of a greater local immune reactions. Ahmed et al. retrospectively analyzed data from two prospective nivolumab trials in patients with advanced disease treated at a single institution, selecting patients with BMs who were treated with SRS within 6 months of receiving nivolumab. Local brain control rate was 91 and 85%, respectively, at 6 and 12 months, with a median survival time of 12 months and a distant BM control rate of 53%. These preliminary results suggest a better intracranial control with nivolumab compared with ipilimumab, probably related to a higher clinical activity and a lower toxicity profile; however, these initial findings should be confirmed in prospective trials (265). Combinations of ipilimumab and new molecular agents, such as trametinib (MEK inhibitor), are under investigation (266).

Dabrafenib and vemurafenib are potent kinase inhibitors of BRAF V600E-mutated melanoma cells, with substantial activity in BRAF-mutated melanoma BM (267). The activity of dabrafenib in BMs was demonstrated in a large multicenter, open-label, phase II trial which enrolled V600E-V600K-mutated-BRAF metastatic melanoma patients and at least one measurable brain lesion. An overall intracranial objective response rate of 31%, with little difference between patients who progressed after prior CNS therapy and patients who were treatment naïve (243). Dabrafenib was well tolerated, and the number of spontaneous intracranial hemorrhages was lower than those reported after ipilimumab.

The activity of vemurafenib is also meaningful, as both retrospective (268) and phase II trials (269) have reported an intracranial response rate of 16–50%, even if the improvement of OS was disappointing. Acquired resistance to single-agent BRAF inhibitors develops within 6–7 months of therapy (270) and is mainly driven by MAPK reactivation. The combination of a BRAF inhibitor and a MEK inhibitor, in comparison with BRAF inhibitor alone, significantly improved response rates, median PFS/OS in phases I/II (dabrafenib/trametinib vs dabrafenib) (241, 271) and in phase III studies (vemurafenib/cobimetinib vs vemurafenib) (272).

Preliminary clinical reports evaluated the efficacy and safety of RT plus vemurafenib and dabrafenib in patients with BRAF V600E-mutated melanoma BMs (273, 274). These studies indicated a potential synergistic effect, resulting in 75% response rate, 65% of symptomatic relief, and median survival of 13.7 months. Ahmed et al. also showed good local control rates after vemurafenib and SRS with low toxicity (273).

Activating mutations in cKIT have been identified in up to 6% of some cutaneous melanomas subtypes. BRAF wild-type patients, which harbor cKIT mutations, could benefit from TKI inhibitors such as imatinib, an oral cKIT inhibitor that has demonstrated dramatic clinical responses (275, 276).

Dasatinib, a second-generation drug developed for CML (chronic myeloid leukemia) that also acts on cKIT, has better CNS penetration and perhaps more toxicity than imatinib.

### Supportive Care

#### Corticosteroids

Corticosteroids are used to control cerebral edema and mass effect. Two evidence-based guidelines on the role of steroids have been published in Europe (277) and US (278). Dexamethasone is recommended for patients who are symptomatic, with a starting dose of 4–8 mg/day up to higher doses of 16–32 mg/day in patients with severe symptoms. Dexamethasone is the steroid of choice because of its minimal mineral corticoid effect and long half-life, although any other corticosteroid can be effective if given in equipotent doses. A neurological improvement within 24–72 h after beginning of treatment is seen in up to 75% of patients. When used as the sole form of treatment, dexamethasone produces about 1 month’s remission of symptoms and slightly increases the 4- to 6-week median survival of patients who receive no treatment at all (278). To minimize side effects from chronic dexamethasone administration, including proximal myopathy, tapering of steroid dosing within 1 week of starting therapy and discontinuation within 2 weeks is encouraged. By contrast, asymptomatic patients do not need corticosteroids, even during radiotherapy.

The need for anticonvulsant medication is clear in patients who have experienced a seizure by the time their brain tumor is diagnosed. The evidence does not support prophylaxis with antiepileptic drugs (AEDs) in patients with brain tumors, including metastases. Twelve studies, either randomized trials or cohort studies, investigating the ability of prophylactic AEDs (phenytoin, phenobarbital, and valproic acid) to prevent first seizures, have been examined, and none have demonstrated efficacy (279). Subtherapeutic levels of anticonvulsants were extremely common and the severity of side effects appeared to be higher (20–40%) in brain tumor patients than in the general population receiving anticonvulsants, probably because of drug interactions. Phenytoin, carbamazepine, phenobarbital and to a lesser extent oxcarbazepine stimulate the cytochrome P450 system and accelerate the metabolism of corticosteroids and chemotherapeutic or targeted agents, such as nitrosoureas, paclitaxel, cyclophosphamide, topotecan, irinotecan, thiotepa, adriamycin, methotrexate, imatinib, erlotinib, and other TKIs, and thus reduce their efficacy. The role of prophylactic anticonvulsants remains to be addressed in some subgroups of patients, who have a higher risk of developing seizures, such as those with metastatic melanoma, hemorrhagic lesions, and multiple metastases (277, 280). A recent meta-analysis in patients with BM concluded that primary prevention with AEDs might not reduce the risk of seizures, and it is associated with frequent adverse effects (281). For patients who underwent a neurosurgical procedure the efficacy of prophylaxis has not been proven. The efficacy of newer AEDs (levetiracetam, topiramate, gabapentin, lamotrigine, lacosamide, and perampanel) has not been extensively investigated but in some retrospective studies their use in patients with seizures related to BMs, significantly reduce seizure frequency (independently of systemic treatment), produce few side effects and appear not to affect life expectancy (282).

#### Anticoagulation

Anticoagulation is the standard treatment for acute venous thromboembolism (VTE) in cancer patients. The ASCO published updated evidence-based guidelines for the treatment and prevention of VTE in patients with cancer based on a systematic review of the literature. These guidelines address the treatment and prevention of VTE in hospitalized medical and surgical cancer patients and in ambulatory patients receiving cancer therapy. They also concern immediate and extended secondary prophylaxis in patients with established VTE, the potential role of anticoagulation in the treatment of patients with cancer without other recognized indication, and the importance of VTE risk assessment in cancer patients [(283), https://www.asco.org/sites/new-www.asco.org/files/content-files/practice-and-guidelines/documents/VTE-Summary-of-Recs.pdf].

LMWH is preferred over UFH for the initial 5–10 days of anticoagulation for the cancer patient with a newly diagnosed VTE who does not have severe renal impairment (defined as creatinine clearance <30 mL/min). For long-term anticoagulation, LMWH for at least 6 months is preferred due to improved efficacy over vitamin K antagonists. Prolongation of anticoagulation over 6 months has to be considered for select patients with active cancer, such as metastatic disease or those receiving chemotherapy. The insertion of an inferior vena cava filter is only indicated for patients with contraindications to anticoagulant therapy or as an adjunct to anticoagulation in patients with progression of thrombosis (recurrent VTE or extension of existing thrombus) despite optimal therapy with LMWH (284).

In patients with CNS malignancies and VTE anticoagulation is recommended as described for other patients with cancer. Careful monitoring is necessary to evaluate the risk of intracerebral hemorrhage on one side and, on the other, the effect of anticoagulation on survival. These patients merit individualized discussions of the risk and benefit of anticoagulation therapy (284, 285).

Use of novel oral anticoagulants for either prevention or treatment of VTE in cancer patients is not recommended at this time. An open-label, non-inferiority trial, randomly assigned patients with cancer and acute symptomatic or incidental VTE to receive either low-molecular-weight heparin followed by oral edoxaban at a dose of 60 mg once daily (edoxaban group) or subcutaneous dalteparin at a dose of 200 IU/kg of body weight once daily for 1 month followed by dalteparin at a dose of 150 IU/kg once daily (dalteparin group) for 6–12 months. This study concluded that oral edoxaban was non-inferior to subcutaneous dalteparin with respect to the composite outcome of recurrent VTE or major bleeding. The rate of recurrent VTE was lower but the rate of major bleeding was higher with edoxaban than with dalteparin (286).

Although several new studies are ongoing, some important questions remain regarding the relationship between thrombosis and cancer and the optimal care of patients at risk for VTE, in particular with CNS malignancies.

## Executive Summary

• Advances in chemotherapy and targeted therapies have improved survival in cancer patients with an increase of the incidence of newly diagnosed BMs representing a stimulating challenge for development of new therapies.
• Advanced neuroimaging techniques have been increasingly used in the detection, treatment planning, and follow-up of BM.
• Better understanding of mechanisms of brain metastatization and molecular characterization of BM could help finding more selected and effective targeted therapies.
• More sophisticated methods of tumor analysis, including detection of circulating biomarkers in fluids (liquid biopsy), have shown promising information regarding tumor treatment response and progression.
• The therapeutic choice is guided by prognostic index, reserving surgical approaches to the subgroup of patients with controlled systemic disease and good performance status. Radiosurgery of the resection cavity may offer comparable survival and local control as postoperative WBRT. WBRT alone is now the treatment of choice for patients with single or multiple BMs not amenable to surgery or radiosurgery, or with poor prognostic factors, also considering the neurocognitive sequelae of WBRT. Technological advances, such as IMRT with hippocampal sparing and pharmacological approaches (memantine and donepezil), could reduce the risk of cognitive sequelae.
• Leptomeningeal disease (LMD) can be a complication, especially in posterior fossa metastases undergoing a “piecemeal” resection.
• In the last decades, a multitude of molecular abnormalities have been discovered representing potential druggable alterations, such as mutations of EGFR, ALK in NSCLC and HER2-mutations in breast cancer. Novel targeted and immunotherapeutic agents have also revolutionized the systemic management of melanoma.

## Author Contributions

FF contributed to the revision of the literature and to the writing of the manuscript with input from all authors revised. RR and RS supervised development of work and helped in data interpretation and manuscript final revision.

## Conflict of Interest Statement

The authors declare that the research was conducted in the absence of any commercial or financial relationships that could be construed as a potential conflict of interest.
